# Life-threatening haemolysis in a patient with acute copper sulphate poisoning

**DOI:** 10.4103/0019-5049.79878

**Published:** 2011

**Authors:** Nishant Sood, PK Verma

**Affiliations:** Department of Anaesthesiology & Intensive Care, Vardhman Mahavir Medical College & Safdarjang Hospital, New Delhi - 110 029, India

Sir,

A 29-year-old male with copper sulphate poisoning presented with severe haemolysis that made blood grouping and other laboratory parameters difficult to determine. The patient had earlier been admitted in another hospital with symptoms of nausea, vomiting, diarrhoea, melena and haematuria. Investigations there revealed haemoglobin (Hb) 8.8 g%, serum bilirubin 1.4 mg%, serum creatinine 6.8 mg%. There he was managed conservatively; as the patient’s condition continued to deteriorate he was referred to a tertiary care centre for further management. On arrival at our hospital, he was conscious but agitated with pulse rate of 132/min; blood pressure 120/86 mmHg; respiratory rate 42/min. The skin was ‘mauve lavender’ and pulse oximeter readings were 82-85%. The patient was put on mechanical ventilation. Patients Hb further fell down to 2.8 g%. Due to massive haemolysis arterial blood gas analysis (ABG), blood grouping and cross matching, renal and liver function tests could not be done as the machine did not accept the haemolysed sample. As a life saving measure, 3 U of O negative packed red blood cells were transfused. Investigations later in the day revealed Hb 4.5 g%; leucocyte count 24000 cells/cu. mm; platelet count 14200/cu mm; blood urea 92 mg%, serum creatinine 2.8 mg%; total bilirubin 8.3 mg% with 3.6 mg% conjugated; prothrombin time 18.8 s (control 13 s); partial thromboplastin time 42.9 s (control 30 s); international normalized ratio 1.9. ABG revealed pH of 7.12, HCO_3_8.5 and base excess of - 18.2 mEq/L. Serum electrolytes, electrocardiogram and chest radiograph were normal. Blood group was AB positive. Peripheral smear was positive for Heinz bodies. Urinary free haemoglobin was positive.

The patient received two additional units of O negative packed red cells and 2 U of fresh frozen plasma. Sodium bicarbonate was administered to correct acidosis and adequate hydration was maintained. Methylene blue was infused; a total dose of 4 mg/kg was given over 6 h. Facilities for measurement of methaemoglobin levels and co-oximetry are not available at our institution.

On day 2, the patient remained haemodynamically stable; there was no melena or haematuria. Weaning from mechanical ventilation was initiated on day 2 of hospital stay and the trachea was extubated on day 3. The patient was transferred to the medical ward on day 4 and was discharged home on day 12.

Following copper ingestion, gastrointestinal symptoms generally develop within 15 min and may be severe enough to produce shock. Early deaths are due to shock, while hepatic and renal failure account for delayed mortality. Copper poisoning affects the erythrocytes, the liver, and the kidneys in the order named. Intravascular haemolysis appears 12-24 h following ingestion. Haemolytic anaemia is caused either by direct cell membrane damage or indirectly as a result of the inactivation of enzymes which protect against oxidative stress.[1,2] Jaundice is partly hepatic in origin in addition to haemolysis.[3] The haem pigment released due to haemolysis, direct toxic effect of copper, and hypotension as a result of gastrointestinal losses may result in renal failure. When the concentration of hepatic copper is greater than 50 mg/g dry weight, liver cell necrosis occurs with release of large amounts of copper into the serum. This released copper is taken up by the erythrocytes and may account for the delayed secondary episode of haemolysis.[1] Our patient probably had delayed haemolytic anaemia as evidenced by a fall in haemoglobin concentration from 8.8 g% at the time of admission to the private hospital to 2.8 g% on admission to our hospital.

Copper poisoning is associated with high mortality compared to other metal toxicities.

Treatment of copper sulphate poisoning is largely symptomatic. Though chelating agents have been used in the treatment of acute copper poisoning,[4] there are no controlled studies regarding their use.[1,5] In addition, the presence of acute renal failure limits the potential for antidotes. The role of dialysis is limited to the management of associated renal failure. We did not use chelators in our case. The role of steroids to treat corrosive burns is controversial.[1] Hepatic and renal dysfunction in our case was managed conservatively.
